# Rural health research in the 21st century: A commentary on challenges and the role of digital technology

**DOI:** 10.1111/jrh.12903

**Published:** 2024-12-16

**Authors:** Mairead Moloney, Jasmine Rubio, Israel Palencia, Laronda Hollimon, Dunia Mejia, Azizi Seixas

**Affiliations:** ^1^ Department of Informatics and Health Data Science Miller School of Medicine, University of Miami Miami USA; ^2^ Frost Institute for Data Science and Computing, University of Miami Miami USA; ^3^ Media and Innovation Lab, Miller School of Medicine, University of Miami Miami USA

**Keywords:** digital equity, digital technology, rural health research, rural population

Rural health research, fundamental to US public health, has faced significant challenges including inconsistencies in defining rural areas, methodological constraints in studying dispersed populations, and complex social and cultural factors.^1^, ^2^ This commentary reexamines these enduring issues and proposes innovative solutions leveraging digital technologies. While acknowledging the potential of these technological approaches, we also address barriers to digital equity in rural settings and suggest practical strategies to overcome them.

## DEFINITIONAL INCONSISTENCY

Defining “rural” poses significant challenges.^1^, ^2^ Current classification methods typically consider population density, proximity to urban centers, and infrastructure availability. However, these approaches often lead to inconsistencies.^1^, ^3^ The US Census, for instance, identifies urban areas based on population density, with non‐urban areas classified as rural.^1^ This method, while systematic, often overlooks crucial factors like commuting patterns, employment nature, land use, and access to essential services—including internet connectivity and advanced medical care.

Online tools have emerged to address these limitations by incorporating multiple definitions of rurality. The Rural Health Information Hub's “Am I Rural?” tool exemplifies this approach, integrating seven distinct definitions including data from the US Census, Rural‐Urban Commuting Areas, and Federal Office of Rural Health Policy classifications.^4^ This tool also considers federal grant eligibility and health care professional shortages, providing a more comprehensive assessment of rural status.

The “Am I Rural?” tool illustrates how technological advancements can enhance rural area definition precision.^4^ By employing a multifaceted approach, these tools enable more accurate representations of rurality in health research. Consequently, this can inform policy decisions and resource allocation more effectively, ultimately benefiting rural communities' health and wellness.

## METHODOLOGICAL CONCERNS

Smaller population size, low population density, and limited access to transportation often pose methodological challenges for rural participant recruitment and retention, particularly if in‐person data collection is required.^2^, ^5^ Additionally, research questions or scales that are urban‐normative (i.e., urban lifestyles or values are viewed as the default/ideal) may alienate respondents, leading to reduced response rates and questionable validity.^2^ Dissemination of rural research findings is often more challenging due to confidentiality concerns in smaller communities.^6^, ^7^

To address these challenges, researchers are increasingly turning to innovative digital approaches. For instance, Vos et al.^8^ created a standalone page on a popular social media platform resulting in successful engagement and recruitment of rural participants. This low‐cost, accessible method was particularly impactful when researchers’ community engagement was highlighted. Once recruited, real‐time interactions via text messages or Zoom, or data collection reminders via mobile applications (“apps”) offer personal, timely, and tailored communications. Rural residents feel positively about personal technology, including apps and wearables, and view technology as a means of bridging resource gaps.^9^, ^10^

Health assessment and intervention tools designed for urban populations may be adapted for rural participants using community‐based participatory research (CBPR). CBPR, where community members and key informants actively collaborate with researchers, has long been used in rural communities. However, integrating CBPR with health informatics research has shown distinct benefits, including increased recruitment of diverse populations, improved internal validity, and more rapid translation of research into action.^11^ CBPR has also proven helpful in identifying best practices in dissemination of research findings in small communities.^12^ Research participants have identified helpful digital tools including: digital animation; Facebook live sessions; short text messages, email, videos, podcasts; and/or social media campaigns, as creative and engaging ways to disseminate findings while maintaining confidentiality.^6^, ^7^ Moreover, employing interactive methods such as gamification and chatbots can enhance the dissemination of health‐related content and the promotion of health education.

## SOCIAL/CULTURAL ISSUES

Rural communities have always been diverse, with residents from varied cultural, ethnic, and socioeconomic backgrounds.^13^ Recent demographic shifts, including an increased presence of immigrants from Spanish‐speaking countries, are further diversifying rural America.^13^ However, this diversity has often been overlooked and may be complicated by strong insider/outsider dynamics common in rural communities.^2^ These dynamics can create challenges for health researchers perceived as outsiders.^2^

Rural health researchers must first understand the complexity of rural socio‐cultural dynamics and additionally avoid oversimplified recruitment and retention approaches. Here too, digital technology may play a key role in effective engagement with diverse rural populations. Social media (e.g., Facebook) and Global Positioning System‐based apps have been found effective in attracting and retaining more diverse participant samples.^14^ However, the literature is clear that digital connections alone are insufficient to overcome community exclusion practices. Best practices are to use technology in a CBPR framework as well as rely on community‐based recruiters (aka “community leaders” or “champions”).^14^, ^15^, ^16^ Bilingual and ethnically diverse research staff who share participants’ cultural identity may enhance community acceptance as well as participant experience.^17^, ^18^

Further, researchers must be careful to attend to challenges including complex, culturally sensitive health topics.^18^ Considering participants’ specific national, cultural, and geographic context is vital to culturally competent research. Important factors to consider include: national origin, migration patterns (e.g., nearby vs. distant countries, family vs. individual migration), length of US residence, acculturation levels, education and socioeconomic status, and social context of settlement areas (including local attitudes towards immigrants, regional legislation, and access to education).^19^

## DIGITALLY UNDERSERVED POPULATIONS/DIGITAL DIVIDE

Although digital technologies may offer creative, feasible, and effective solutions to longstanding rural research challenges including definitional inconsistency, methodological issues, and social/cultural norms, they also risk deepening preexisting inequalities.^20^ Technology barriers including design, access, and digital literacy must be adequately addressed to close the health disparity gap in rural regions, and beyond.

### Design

Digital health technology designs should focus on prioritizing inclusivity and accessibility for diverse populations, particularly in underserved rural communities.^21^, ^22^ A study by Wu et al. found that most health technology applications use user‐centered design to tailor user experience.^21^ Engaging and soliciting feedback from populations who are diverse in age, culture, and ethnicity, as well as sensory and intellectual abilities, is key to the tailoring process. It is also essential to employ iterative processes, incorporating multiple surveys/interviews/focus groups throughout the process to collect user feedback at time points where adjustments may be made.^21^ However, particularly in fast‐changing rural spaces, researchers and health technology developers should also consider oft‐neglected criteria including participants’ education level, profession types/labor demands, cultural mistrust in government entities, language, and immigration status.

### Access

US internet access, considered a social determinant of health, is inherently inequitable and disproportionately impacts rural regions.^23^ Two factors influencing this disparity are cost and infrastructure. In 2021, the federal government implemented the Affordable Connectivity program (ACP)^24^ to lower the price of internet services by giving income‐eligible and tribal land consumers monthly discounts. Unfortunately, the ACP ended in 2024 leaving many unable to afford access.^25^, ^26^ However, another government program called Lifeline^27^ helps individuals and families on government assistance obtain telephone service (landline, mobile) broadband services and devices (mobile phone) for a discount.

Recent federal programs have also sought to address the issue of infrastructure. In 2021 and 2022 states received money from the Broadband Equity, Access, and Deployment (BEAD) program.^14^, ^15^ This program helps state governments deploy, map, and expand broadband services in all 50 states, DC, and territories. The USDA ReConnnect program, a loan and grant program, helps expand broadband services and deployment in rural communities to organizations and companies.^28^ While these programs are poised to increase internet access in many underserved, rural communities it is imperative that state and federal governments recognize the digital divide as key to health disparities and provide resources accordingly.^29^

### Digital literacy

In addition to broadband internet access, digital literacy is crucial for rural health research. Digital literacy encompasses different skill sets, cognitive abilities and technologies such as using computers, smart phones, applications, internet navigation and spotting misinformation.^30^ The US demographic group least likely to be digitally literate or own digital technology is older adults (ages 55+).^31^ Unfortunately, the population is aging more rapidly in rural areas, compared to urban areas.^32^

To promote health equity across geographies and lifespan, health researchers must actively recruit and engage with rural older adults. To ensure that these research participants are comfortable with digital data collection methods (e.g., online surveys, Fitbits) researchers may wish to bridge the gap with more “analog” methods. For instance, participants may appreciate a scheduled phone call to walk them through the setup and management of technology. Encouraging participation in local digital literacy programs, such as the Digital Connectors program or Public Library Association (PLA) courses, can also help address these challenges and improve the quality of rural health research.^33^ DigitalLearn.org, the cornerstone of the PLA program, offers self‐directed, tailored online materials in both English and Spanish.^30^

## CONCLUSIONS

Rural health research faces persistent challenges including definitional inconsistency, methodological issues, and cultural barriers. While digital technology offers promising solutions to enhance rural research, particularly in a time of increasing population diversity, it also introduces new considerations such as access and digital literacy. As we advance into the 21st century, addressing these challenges and leveraging technological opportunities thoughtfully will be crucial. The future of rural health research depends on our ability to adapt, innovate, and prioritize the unique needs of rural communities, ensuring their voices are central in shaping health research and policy.

## CONFLICT OF INTEREST STATEMENT

The authors declare no conflicts of interest.
